# Characterization of *n*-Hexane sub-fraction of *Bridelia micrantha *(Berth) and its antimycobacterium activity

**DOI:** 10.1186/1472-6882-11-28

**Published:** 2011-04-11

**Authors:** Ezekiel Green, Lawrence C Obi, Amidou Samie, Pascal O Bessong, Roland N Ndip

**Affiliations:** 1 AIDS Virus Research Laboratory, Department of Microbiology, University of Venda, Thohoyandou 0950, South Africa; 2 Department of Biochemistry and Microbiology, University of Fort Hare, Private Bag X1314, Alice 5700, South Africa; 3 Research and Academic Directorate, Walter Sisulu University, Mthatha, South Africa; 4 Department of Biochemistry and Microbiology, University of Buea, Cameroon

## Abstract

**Background:**

Tuberculosis, caused by *Mycobacterium tuberculosis *(MTB), is the most notified disease in the world. Development of resistance to first line drugs by MTB is a public health concern. As a result, there is the search for new and novel sources of antimycobacterial drugs for example from medicinal plants. In this study we determined the *in vitro *antimycobacterial activity of *n*-Hexane sub-fraction from *Bridelia micrantha *(Berth) against MTB H_37_Ra and a clinical isolate resistant to all five first-line antituberculosis drugs.

**Methods:**

The antimycobacterial activity of the *n*-Hexane sub-fraction of ethyl acetate fractions from acetone extracts of *B. micrantha *barks was evaluated using the resazurin microplate assay against two MTB isolates. Bioassay-guided fractionation of the ethyl acetate fraction was performed using 100% *n*-Hexane and Chloroform/Methanol (99:1) as solvents in order of increasing polarity by column chromatography and Resazurin microtiter plate assay for susceptibility tests.

**Results:**

The *n*-Hexane fraction showed 20% inhibition of MTB H_37_Ra and almost 35% inhibition of an MTB isolate resistant to all first-line drugs at 10 μg/mL. GC/MS analysis of the fraction resulted in the identification of twenty-four constituents representing 60.5% of the fraction. Some of the 24 compounds detected included Benzene, 1.3-bis (3-phenoxyphenoxy (13.51%), 2-pinen-4-one (10.03%), N(b)-benzyl-14-(carboxymethyl) (6.35%) and the least detected compound was linalool (0.2%).

**Conclusions:**

The results show that the *n-*Hexane fraction of *B. micrantha *has antimycobacterial activity.

## Background

Healthcare burden resulting from an estimated 13.3 million prevalent cases of tuberculosis (TB) and 2.32 million deaths [1] has been made worse by the emergence of multidrug-resistant TB (MDR-TB). Although tuberculosis is treatable, few alternative drugs are available in cases where drug resistance is a problem. Second-line drugs such as kanamycin, *p-*aminosalicylate, ethionamide and fluoroquinolones currently used are either less effective or toxic [1]. There is therefore an urgent need to discover and develop new anti-TB drugs to target drug resistance, and improve the treatment of latent TB [2].

Natural products isolated from terrestrial plants have played a major role in the discovery of drugs against infectious diseases [3]. Approximately 10% of the world's terrestrial plants, some being used medicinally, are found in South Africa. However, few of these plants have been investigated for anti-TB activity, yet TB is one of South Africa's biggest healthcare problems. In a preliminary screening of selected South African plants for antimycobacterial activity, we observed that the acetone extract of *B. micrantha *barks showed potent growth inhibition of MTB with a MIC of 25 μg/mL [4].

*B. micrantha *(*Euphorbiaceae*) is commonly used traditionally for gastro-intestinal ailments, painful joints, retained placenta, diabetes mellitus, syphilis, prehepatic jaundice, tape worm abdominal pain, conjunctivitis, headache, scabies, bloody diarrhoea, dysentery, emetic, wound infection, coughs, threadworms, tonic for children, sore eyes, epigastric pain, relief of headache, purgative diarrhoea and worms [5-7]. Essential oils have long been used for a wide variety of medicine [8]. Antimicrobial properties of essential oils have been well documented [9,10]. Compounds found in *Anethum graveolens *have been reported to have various degrees of antimycobactetial activity [11].

In this study, we report on the effects of the *n*-Hexane fraction, obtained from acetone extract of *B. micrantha*, on the growth of mycobacteria. Furthermore, we characterized constituents from this active fraction using GC/MS.

## Methods

### Plant material, extraction and purification

The acetone extracts of *B. micrantha *barks were prepared following a previously reported scheme [12]. Briefly, the air dried barks of the plant were pulverized and extracted three times by maceration in acetone for 72 h at room temperature. The solvent extract was evaporated to yield a brownish viscous residue. The acetone extracts were further extracted using ethyl acetate. The ethyl acetate fraction (9 g) was further fractionated using Gravity column chromatography with 100% *n*-Hexane, 1% Methanol in Chloroform in order of increasing polarity up to 5% Methanol in Chloroform, then 100% Methanol to yield sixteen sub-fractions of increasing polarity (F1-F16). Each sub-fraction was tested for activity against MTB H_37_Ra and the resistant isolate [13].

### Mycobacteria used

MTB strain H_37_Ra (American Type Culture Collection 27294) and an MDR clinical isolate were used in the present study. The first (H_37_Ra) is sensitive and the second (a local isolate) is resistant to all five first-line antituberculosis drugs (streptomycin, isoniazide, rifampin, ethambutol, and pyrazinamide). The clinical isolate was isolated, identified, and characterized in the Mycobacteriology Laboratory at the University of Limpopo, MEDUNSA Campus, Pretoria, South Africa from a patient with advanced pulmonary tuberculosis [14].

### Antituberculosis activity

Antimycobacterial bioassay was performed using the Resazurin microplate assay (REMA) [15]. The fractions were dissolved in DMSO to give a concentration of 1 mg/mL. Control experiments with organism and decreasing concentration of DMSO were performed to avoid DMSO affecting the toxicity of the fractions to MTB. Suspensions of MTB H_37_Ra strains were prepared at a concentration of about 10^5 ^cells/mL. One hundred microlitre of the bacterial suspension was added to each well of a microtiter plate together with the plant fraction in Middlebrook 7H9 broth to a final volume of 200 μL, and the final concentration of the *n*-Hexane fraction ranged from 0.391 to 100 μg/mL. After incubation for 7 days, 20 μg/mL of Resazurin dye was added to the control well. If the dye turned pink, indicating bacterial growth, the dye was then added to all remaining wells in the plate. The results were read the following day using a microtiter plate reader (Bio-Rad 680, South Africa). The same drug sensitivity procedure was applied to the MDR isolate. For standard tests, the MIC values of rifampin and isoniazid were determined each time. The acceptable MIC ranges of the drugs were 0.0047-0.0095 and 0.05-0.1 μg/mL respectively [16]. The experiments were done in triplicate and the average concentrations were reported.

### Phytochemical analysis of fractions

Chemical constituents of the extracts were analyzed by thin layer chromatography (TLC) using aluminium-backed TLC plates (Merck, silica gel 60 F254). The TLC plates were developed with one of the four eluent systems, ethyl acetate/methanol/water (40:5.4:5): [EMW] (polar/neutral); chloroform/ethyl acetate/formic acid (5:4:1): [CEF] (intermediate polarity/acidic); benzene/ethanol/ammonium hydroxide (90:10:1): [BEA] (non-polar/basic) [17], Hexane/Ethyl acetate (150:1) [HE] (non-polar). Development of the chromatograms was done in a closed tank in which the atmosphere had been saturated with the eluent vapour by lining the tank with filter paper soaked with the eluent [18].

### TLC analysis of the fractions

Visible bands were marked under daylight and ultraviolet light (254 and 360 nm, Camac Universal UV lamp TL-600) before spraying with freshly prepared vanillin (0.1 g vanillin, 28 mL methanol, 1 mL sulphuric acid) spray reagents [19]. The plates were carefully heated at 100°C for optimal colour development. Fraction (**F1**) (200 mg) (100% *n*-Hexane fraction) and **F5 **(150 mg) (1.75% Methanol in Chloroform) were identified as putative pure compounds because only one band was observed on TLC plates.

### GC-MS analyses and identification of components

The GC-MS analyses was carried out using Hewlett-Packard HP 5973 mass spectrometer interfaced with an HP-6890 gas chromatograph with an HP5 column (30 m × 0.25 mm id, 0.25 μm film thickness) and an MS detector. The oven temperature was programmed from 70°C (after 2 minutes) to 325°C at 4°C/min, final temperature was held for 10 minutes at 240°C. The ion source was set at 240°C and electron ionization at 70 Ev. Helium was used as the carrier gas (1 mi/min). The split ratio was 1:25 with the scan range of 35 to 425 amu. Hexane fraction (1.0 μL), diluted in hexane was manually injected into the GC/MS. The components of the oils were identified based on the comparison of their retention indices and mass spectra with the standards, the Wiley 275 Library of Mass Spectra database (Wiley, New York) of the GC/MS system and published data [19-21].

## Results

### Plant material, extraction and purification

Nine grams of ethyl acetate fraction were collected from 30.412 g acetone extracts, showing 29.6% yield. The *n*-Hexane fraction yields were calculated on a dry weight basis as 0.5% (0.045 g/9 g × 100) and was analysed to determine its constituents (Table 1). In order to identify putative active compounds present within *B. micrantha n*-Hexane fraction, we employed a gas chromatography/mass spectrometry (GC/MS) system. Twenty-four derivatives were present whose abundance was more than 0.1%, corresponding to N (b)-benzyl-14-(carboxymethyl), Benzene, 1,3-bis (3-phenoxyphenoxy), 2-Pinen-4-one as major compounds. All the compounds identified are shown in Table 1. **F5 **was identified as 3-oxo-11α-hydroxyolean-12-ene-30-oic acid when compared with the standards on TLC.

**Table 1:** The phytochemicals of the *n*-Hexane fractions from acetone extracts of *Bridelia micrantha*

Peak No	Retention Time (Min)	**MS + T**_**ret **_identification^*^	*%
1	3.19	N(b)-benzyl-14-(carboxymethyl)	6.35
2	3.21	Benzene, 1.3-bis (3-phenoxyphenoxy)	13.51
3	3.43	2-Phenyl-2-tipyl-acenapthenone	4.50
4	5.96	α-pinene	0.49
5	6.30	Camphene	0.14
6	8.28	1.8 cineole	0.91
7	11.32	Camphor	2.77
8	11.91	Endo-Borneol	1.76
9	12.23	Linalool	0.20
10	12.60	1-α-Terpineol	0.43
11	12.88	α-Caryophyllene oxide	0.95
12	12.94	Nopol	0.45
13	13.10	2-Pinen-4-one	10.03
14	15.12	(-)-Bornyl acetate	1.96
15	17.81	1 Tetradecanol (Fatty alcohol)	1.07
16	22.60	5-Octadecene	1.10
17	29.64	Hexadecanoic acid (Methyl ester)	1.02
18	30.38	Palmitic acid	3.91
19	34.09	17-Pentatriacontene	1.51
20	36.27	Tritetracontane	1.36
21	36.88	5.beta.-Pregn-11-ene	1.71
22	38.76	4-Imidozolidinone	3.49
23	40.52	Naphthalene	1.06
24	40.82	Quinoline	1.58
TOTAL			60.55%

*Only the percentages over 0.1% are indicated.

### Antituberculosis activity

The acetone extracts of *B. micrantha *that showed activity on MTB isolates [4] was submitted to bioassay-guided fractionation using column chromatography. Primary fraction F1 (ethyl acetate fraction) showed antimycobacterial activity on MTB H_37_Ra. F1 was further fractionated as mentioned above and showed an MIC value of 8.25 μg/mL against H_37_Ra MTB strain. The fraction also inhibited the growth of the local MTB isolate resistant to INH, STM, EMB, and RIF at a concentration of 50 μg/mL. Statistical significance was observed only when **F1 **was compared with first line antibiotics (Odd ratio [OR] = 1.84, 95% CI 1.37-2.46, p value for trends = 0.0012). The final concentration of DMSO (2%) in the broth did not show any inhibition of MTB.

## Discussion

Plants have been used throughout history in traditional medicines for the treatment of diseases worldwide. Today, approximately two-thirds to three-quarters of the world's population are estimated to rely on medicinal plants as their primary source of medicines [22]. There has been an increasing interest in studying the biological properties of plants and their derivatives for discovering biologically active compounds [23,24]. Phytochemicals with prominent pharmacological properties which were not previously identified were discovered from plant product [25]. They have been defined as small organic biomolecules generally hydrophobic and designated as naturally occurring antibiotics [26]. Coagulation of cytoplasmic membrane, breakdown of proton motive force, breakdown of electron flux and the cause of imbalance in active transport are some of the antimicrobial mechanisms of phytochemicals [26].

The hexane fractions are known to contain essential oils. Several studies have demonstrated that essential oils hold therapeutic value in treatment of diseases and are well tolerated [27-29]. The Hexane fractions in our study demonstrated 80% inhibition at 30 μg/mL on clinical isolate resistant to RIF, EMB, INH and STM and more than 90% on MTB H_37_Ra.

In many essential oils, the antimicrobial activity is due to the presence of isoprenes such as monoterpenes, sesquiterpenes or related alcohols and phenols [30]. The activity of *n*-Hexane fraction of *B. micrantha *is probably due to the presence of isoprenes identified by GC/MS in our study. Monoterpenes have been shown to poses antimycobacterial effect at lower (100 μg/mL) concentrations [31]. More specifically α-pinene was shown to have a MIC of 64 μg/mL against MTB H_37_Rv [32].

It was demonstrated that the physical properties of essential oils significantly influences the actions of the individual components, increasing or reducing antimicrobial efficacy [32]. The hydrophilic character of monoterpenes functional groups and the lipophilic character of their hydrocarbon skeleton are of main importance in the antimicrobial action of essential oil components [33]. The disturbance of lipid fraction of the plasma membrane could be the antibacterial activity of terpenes, resulting in alterations of membrane permeability and leakage of intracellular materials [34]. This effect may also be a consequence of the interaction between the major and minority components of essential oil. Essential oils need to be diluted in alcohols that enhance their volatility and aroma. In determining where the antimicrobial effect of *n*-Hexane fraction is derived from, we tested 2% DMSO on both MTB isolates. The antimycobacterial effect was found to be due to essential oil as 2% DMSO did not inhibit growth of MTB.

*B. micrantha *stem bark was reported to be abortifacient with potentially toxic effects probably due to the presence of delphinidin and methyl salicylate [35]. Those two compounds were not observed among the isolated essential oils in our study. The major components of the essential oil in the present study were Benzene, 1.3-bis (3-phenoxyphenoxy) (13.51%), and 2-Pinen-4-one (10.13%). Antimycobacterial activities of essential oils with 1,8 Cineole, camphor, α-Pinene, borneol as major compounds were recently elucidated [36]. Although their percentage is low, the same compounds were found in the *n*-Hexane fraction in our study. In a study by Lamproti and co-workers [37], α-pinene, α-terpineol, caryophyllene, were found to inhibit the proliferation of tumor cells *in vitro*.

## Conclusions

The essential oils and volatile compounds may provide an important source of new antimycobacterial agents. However, toxicity of these complex mixes should be determined.

## Abbreviations

MTB: *Mycobacterium tuberculosis*; INH: Isoniazid; EMB: Ethambutol; STM: Streptomycin; RIF: Rifampicin.

## Competing interests

The authors declare that they have no competing interests.

## Authors' contributions

EG conceived the study, developed the design, collected plants, carried out the experiments, and drafted the manuscript. AS collected plants, dried and ground them, performed the extraction for screening and participated in the manuscript preparation and revision, LCO, POB and RNN participated in the concept and design of the study, supervised the work, data analysis and interpretation and preparation of the manuscript. All authors read and approved the manuscript.

## Pre-publication history

The pre-publication history for this paper can be accessed here:

http://www.biomedcentral.com/1472-6882/11/28/prepub
